# Intratumoral Pi deprivation benefits chemoembolization therapy via increased accumulation of intracellular doxorubicin

**DOI:** 10.1080/10717544.2022.2081384

**Published:** 2022-05-30

**Authors:** Yang-Feng Lv, Zhi-Qiang Deng, Qiu-Chen Bi, Jian-Jun Tang, Hong Chen, Chuan-Sheng Xie, Qing-Rong Liang, Yu-Hua Xu, Rong-Guang Luo, Qun Tang

**Affiliations:** a School of Public Health, Jiangxi Provincial Key Laboratory of Preventive Medicine, Nanchang University, Nanchang, China; b Institute for Advanced Study, Nanchang University, Nanchang, China; c Department of Oncology, The First People’s Hospital of Fuzhou, Fuzhou, China; d Department of Respiratory and Critical Care Medicine, The First Affiliated Hospital of Nanchang University, Nanchang, China; e Department of Interventional Radiology, Jiangxi Province Chest Hospital, Nanchang, China; f Department of Medical Imaging and Interventional Radiology, The First Affiliated Hospital of Nanchang University, Nanchang, China

**Keywords:** Drug resistance, doxorubicin, chemoembolization therapy, hepatocellular carcinoma, sevelamer

## Abstract

It is a decade-long controversy that transarterial chemoembolization (TACE) has definite priority over transarterial embolization (TAE) in treating patients with hepatocellular carcinoma (HCC), since HCC cells are regularly resistant to chemotherapy by enhanced expression of proteins that confer drug resistance, and ABC transporters pump the intracellular drug out of the cell. We addressed this issue by modulating the chemo-environment. In an animal model, sevelamer, a polymeric phosphate binder, was introduced as an embolic agent to induce intratumoral inorganic phosphate (Pi) starvation, and trans-arterially co-delivered with doxorubicin (DOX). The new type of TACE was named as DOX-TASE. This Pi-starved environment enhanced DOX tumoral accumulation and retention, and DOX-TASE thereby induced more severe tumor necrosis than that induced by conventional TACE (C-TACE) and drug-eluting bead TACE (D-TACE) at the same dose. *In vitro* tests showed that Pi starvation increased the cellular accumulation of DOX in an irreversible manner and enhanced cytotoxicity and cell apoptosis by suppressing the expression of ABC transporters (P-glycoprotein (P-gp), BCRP, and MRP1) and the production of intracellular ATP. Our results are indicative of an alternative interventional therapy combining chemotherapy with embolization more effectively.

## 1. Introduction

Hepatocellular carcinoma (HCC) is the fifth most common cancer and the third leading cause of cancer-related deaths worldwide. Cytotoxic chemotherapy has been used for over 30 years, but definite evidence that it prolongs survival has been lacking (Asghar & Meyer, 2012). For example, doxorubicin (DOX) is routinely used as a single drug for advanced HCC patients but has shown ineffectiveness, with a response rate of approximately 15–20%. Other chemotherapy agents, such as oxaliplatin, epirubicin, 5-fluorouracil, etoposide, and their combinations, demonstrate even lower efficacy (Yeo et al., 2005). Despite failure in chemotherapy, some cytotoxic drugs are still being applied for transarterial chemoembolization (TACE), which aims to combine chemotherapy with embolization therapy.

TACE is recommended as one of the most effective treatments for patients suffering from HCC in the intermediate stage (Llovet & Bruix, 2003; Reig et al., 2022), and approximately one million HCC patients received TACE therapy in 2020 in China. By selectively obstructing tumor-feeding arteries with lipiodol mixed with chemotherapeutic agents, TACE induced ischemic necrosis of target tumors. However, whether those chemotherapeutic agents (DOX, oxaliplatin) play a combined therapeutic role is a long-disputing issue. In other words, TACE might not have an advantage over transarterial embolization (TAE) in terms of its comprehensive benefits, including on adverse reactions and overall survival (Marelli et al., 2007; Pleguezuelo et al., 2008; Tsochatzis et al., 2014; Facciorusso et al., 2017). Drug-eluting bead-TACE, emerging as a newly emerging TACE technique, has the unique characteristic of drug release within an elongated time (e.g. months). However, there are still doubts regarding its therapeutic effects according to the clinical outcomes (Song & Kim, 2017).

It is difficult to improve therapeutic effects via modification of the administration route and drug pharmacokinetics. Pharmacological studies have indicated that cytotoxic drugs inhibit the replication of DNA after permeating the cancer cell membrane and entering the nucleus. Therefore, intracellular accumulation is a critical requirement to exert chemotherapy-related cytotoxicity in embolization-induced ischemia. Unfortunately, drug resistance is a major hindrance to the treatment of HCC patients. Cancerous liver cells show enhanced expression of proteins that confer drug resistance. Those proteins, including the transmembrane pump, drive out drugs extracellularly, minimizing their cytotoxicity. Moreover, attempting to overcome that resistance with combinations of additional drugs is highly risky for exacerbating the underlying liver disease (Scudellari, 2014; Lohitesh et al., 2018). The comprehensive benefit from TACE is expected to be better than that from TAE as long as the resistance is reversed or overcome since chemotherapy will synergize with embolization to treat HCC.

Drug resistance is a complex phenomenon that can result from numerous mechanisms. Elevated efflux of anticancer agents by ATP-dependent pumps decreased intracellular drug accumulation, which has been the major reason for resistance of tumors, including HCC, to chemotherapy (Lockhart et al., 2003; Marin et al., 2009; Bar-Zeev et al., 2017; Marin et al., 2020). The resistance caused by abnormally high rates of drug efflux could be either intrinsic or acquired after drug administration. Those pumps responsible for drug efflux are transmembrane transporters, primarily from the ATP binding cassette (ABC) transporter superfamily, such as P-glycoprotein (P-gp; ABCB1; MDR1), breast cancer resistance protein (BCRP; ABCG2), and multidrug resistance-associated protein 1 (MRP1; ABCC1). Specifically, in the case of HCC, all three of these proteins are indicative of HCC progression, and inhibition of their expression contributes to better chemotherapy (Huang et al., 1992; Soini et al., 1996; Nies et al., 2001; Vander Borght et al., 2008; Sukowati et al., 2012; Huang et al., 2013). Targeting these transporters has been proposed for decades but is still far from being applied to the bedside.

As a local-regional therapeutic technique, chemoembolization therapy allows the therapy to be applied to a relatively confined environment of HCC cells. Modulation of the chemo-environment of HCC cells might alter drug resistance; for example, hypoxia-inducible factor (HIF), overexpressed during hypoxia, is primed to mediate metabolic reprogramming during drug resistance in HCC (Bao & Wong, 2021). Moreover, glucose has been proven to sensitize HCC cells to DOX and sorafenib (Chouhan et al., 2020). Our recent work illustrated the transarterial sevelamer embolization (TASE) technique, which promotes intratumoral inorganic phosphate (Pi) starvation (Bi et al., 2022). It is expected that drug resistance might be reversed as Pi starvation induces less ATP production from VX2 cancer cells, since ATP consumption is required to pump the intracellular drug out of the cell. In this work, Pi starvation was found to induce other effects. Pi starvation also led to downregulated expression of the ABC transporter, and both inhibitory effects irreversibly enhanced the intracellular uptake of DOX. In a VX2 animal model, DOX-TASE showed a better therapeutic effect than that of conventional TACE (C-TACE) and drug-eluting bead TACE (D-TACE) when the same dose of DOX was used due to increased accumulation of intracellular DOX.

## 2. Methods and experiments

### 2.1. Implantation of the VX2 rabbit liver cancer model and the general embolization protocol

The protocol for the donor rabbit and the tumor graft procedure are illustrated in Supplemenatry Appendix 1. The general embolization or chemoembolization protocol for the following cohorts is described in Supplemenatry Appendix 2.

### 2.2. Three-cohort treatment for the evaluation of DOX pharmacokinetics, intracellular Pi starvation, ATP synthesis, DOX accumulation, and tumor necrosis

The first cohort was designed to evaluate DOX pharmacokinetics. Nine VX2 rabbits were randomly divided into three groups. In the first group (C-TACE), the total volume of a 0.4 mL emulsion made by mixing 2 mg DOX (MACKLIN, Shanghai, China) in 0.2 mL saline with 0.2 mL lipoidal (Lu-yin Medical Co., Beijing, China) was transarterially administered to individual rabbits. Two milligrams of DOX and 3.5 mg of sevelamer nanoparticles in 0.4 mL iodixanol/saline were transarterially injected into the second group (DOX-TASE). Note that the preparation and physicochemical characterization of sevelamer nanoparticles and their change in morphology after Pi saturation are depicted in Supplemenatry Appendix 3. In the third group (D-TACE), 0.4 mL drug-eluting beads (Calli-Spheres, Hengrui Medical Co., Suzhou, China) with a size range of 100–300 µm containing 2 mg DOX were transarterially administered. At designated intervals (pre-procedure, 2 min, and 60 min after the procedure), blood samples (1 mL) were collected via a catheter inserted into the auricular vein. The DOX concentration in serum was quantified by HPLC–MS according to the protocol described in Supplemenatry Appendix 4. After that, the rabbits were sacrificed, and VX2 tumors were resected to visualize vessel embolization by H&E staining.

In the second cohort, the same grouping and treatment scheme as that used in the first cohort was performed, and the tumor was harvested at different time intervals (2 h and 12 h) after an operation and immediately imaged under the tissue luminescence imaging system (CyTo Bio. IVIS Lumina III, Jiangsu, China). Subsequently, these tumor tissues were broken into two equal parts. Approximately, half of the tumors were used for Pi content determination (Supplemenatry Appendix 5), ATP content determination (Supplemenatry Appendix 6), and analysis of ABC transporter (BCRP; MRP1; P-gp) expression (Supplemenatry Appendix 7). The C-TACE group and DOX-TASE group were compared; the other half of the tumor tissues was immersed in 10% neutral buffered formalin and used for the DOX accumulation assay. The fluorescence accumulation of DOX in tumor cells was expressed as the average intracellular fluorescence intensity = total fluorescence intensity/cell amount.

In the third cohort, a six-armed study was designed to evaluate tumor necrosis. Eighteen VX2 rabbits were randomly divided into three groups (5 mL saline, 1 mL lipoidal, 2 mg DOX in 0.2 mL lipoidal, 2 mg DOX (0.2 mL) in 0.2 mL sevelamer (10 mg/mL in saline), 0.2 mL sevelamer (10 mg/mL in saline), and 0.2 mL drug-eluting bead containing 2 mg DOX (0.2 mL)). The use of seven-day sacrifice time points was done to avoid possible metastasis and invasion based on our previous experience. Harvested tumors were placed into 10% neutral buffered formalin for H&E staining.

Tumor necrosis was used as the primary response outcome measure in all animal studies, given that it is the ultimate appraiser of embolization effectiveness and the pathologic results associated with survival outcomes after all local-regional therapies for HCC. Tumor necrosis – expressed as a percentage of tumor area for each section (%necrosis in the following equation) – was estimated utilizing visual inspection with a manual region of interest generated around the whole tumor and necrotic portions as follows: %necrosis = necrotic area/necrosis whole tumor area × 100. The percentage of tumor necrosis across the three analyzed tumor sections was averaged to provide a tumor necrosis fraction for each tumor. In addition to necrosis quantification, the total fluorescence of DOX distributed inside or within tumors was recorded for each neighboring section.

### 2.3. Pi-dependent intracellular accumulation of doxorubicin and reversibility

Chemicals, reagents, cell lines, and culture conditions are described in Supplemenatry Appendix 8. A laser confocal microscope (Zeiss LSM 880, Oberkochen, Germany) was used to observe the intracellular accumulation of DOX. HEPG-2 cells were plated into confocal dishes and proliferated (approximately 1 × 10^5^ cells) against DOX (1 µM) in media with different concentrations of Pi. After 1 h, the cell culture medium was transferred to a 2 mL centrifuge tube to record the fluorescence emission intensity by fluorescence spectrometry (HORIBA, Longjumeau, France) after centrifugation (12,000 rpm, 10 min).

To record the dynamic flux process, at different time intervals (10 min, 20 min, 40 min, 1 h, 1.5 h, and 2 h), the medium was removed to measure the fluorescence emission of DOX, and the cells were imaged under a laser confocal microscope after they were fixed and stained. In detail, the cells in the confocal dish were washed three times with PBS and fixed in 4% paraformaldehyde for 15 min at room temperature. Next, the cells were washed three times with PBS, and then 4′,6-diamidino-2-phenylindole (DAPI) staining solution (500 µL) was added to the center glass plate for 15 min. Finally, the cells were washed three times with PBS, and intracellular fluorescent signals were visualized under a laser scanning confocal microscope.

The reversibility was tested by incubating the cells in Pi-free medium supplemented with DOX (1 µM) for 1 h and then elevating the Pi concentration to the normal level (125 mg/L) by adding PBS and extending the incubation for an additional 1 h.

### 2.4. DOX-induced cytotoxicity and cell apoptosis under Pi starvation

#### 2.4.1. Cell viability assay

HepG-2 cells were treated with medium with different Pi concentrations (125, 112.5, 93.75, 62.50, 31.25, and 0 mg/L) for 12 h. Then, media with three Pi concentrations, normal (125 mg/L), Pi (–) (31.25 mg/L), and Pi-free (0 mg/L), was prepared, and the cells were incubated in serial concentrations of DOX (0, 0.1, 0.5, 1, 2, 4, and 8 µM) for 48 h. Finally, 10 µL MTT reagent (5 mg/mL, PBS) was added to each well, and the plates were incubated at 37 °C with CO_2_ at 5% and a humidified atmosphere for 4 h. Then, 100 µL DMSO was added to each well after discarding the MTT solution and oscillating at low speed for 10 min on a horizontal shaker. Finally, the absorbance of each well was measured at 490 nm with a spectrophotometric microplate reader (Thermo, Waltham, MA). Cell viability was calculated as a percentage of viable cells versus untreated cells by the following equation: cell viability (%)=(OD (treated) – OD (blank))/(OD (untreated) – OD (blank))×100%.

#### 2.4.2. Apoptosis

HEPG-2 cells (1 × 10^5^ cells) were seeded into six-well plates, and the cells were cultured in four media types (normal Pi, Pi-free, DOX (1 µM) in normal Pi, DOX (1 µM) in Pi-free) for 24 h. Apoptosis was detected using the Annexin V-FITC/PI Kit (4A Biotech, Beijing, China). After being washed with ice-cold PBS and digested with trypsin without EDTA, the cell suspension was collected by centrifugation (1000 rpm × 4 min). Subsequently, the cells were suspended in binding buffer and then incubated with Annexin V (5 µL) and PI labeling solution (10 µL) at room temperature for 15 min in the dark. Ultimately, the percentage of apoptotic cells was measured using a BD Accuri™ C6 Plus Flow Cytometer (BD Biosciences, Franklin Lakes, NJ).

### 2.5. ABC transporter expression by the cells incubated in different Pi concentrations

#### 2.5.1. Real-time PCR (RT-PCR) assay

HepG-2 cells (1 × 10^5^) were cultured in the four media conditions mentioned above for 1 h, and an additional fifth group was incubated in Pi-free medium with DOX (1 µM) for 1 h. Then, the Pi concentration was elevated to the normal level (125 mg/L) for an additional 1 h. Total RNA from cells in these plates was isolated using an RNA Purification Kit (Tiangen, Beijing, China) and reverse transcribed using a Prime Script RT Reagent Kit for RT-PCR (Takara, Otsu, Japan). For quantitative PCR, template cDNA, primers, and TaqMan Gene Expression Master Mix were used according to the manufacturer’s instructions. Three replicate wells were tested per group, and the relative amount of each gene was normalized to the amount of β-actin using the calculation method of 2^–ΔΔt^ and then reported as the fold change at the basal level. The sequences of the primers used for RT-PCR are listed in Supplemenatry Appendix 8.

#### 2.5.2. Western blot (WB) analysis

The incubation conditions in the five groups were the same as mentioned above. Subsequently, the protein expression levels of BCRP, P-gp, and MRP1 in cells were detected by WB; the WB experiment was performed according to the steps described in Supplemenatry Appendix 5.

#### 2.5.3. Immunofluorescence

A total of approximately 1 × 10^5^ HepG-2 cells were cultured in confocal dishes using Pi-free medium or media with normal Pi concentration for 1 h and fixed with 4% paraformaldehyde for 15 min at room temperature. Subsequently, the samples were washed three times with PBS, blocked in 1% BSA at room temperature for 1 h, and incubated with anti-MRP1, anti-MRCP, or anti-P-gp antibodies at 4 °C overnight. The cells were then washed three times with PBS for 10 min each and incubated with an Alexa Fluor-488 secondary antibody at room temperature for 2 h. The cells were stained with DAPI for 10 min and then imaged by laser confocal microscopy (Zeiss LSM 880, Oberkochen, Germany).

### 2.6. Determination of ATP content in HepG-2 cells

HepG-2 cells (1 × 10^5^ cells/well) were plated into six-well plates and then incubated with Pi-free medium for 10 min, 20 min, 40 min, 1 h, 1.5 h, and 2 h. The incubation conditions in the five groups were the same as those mentioned above. The cells were then harvested, and the intracellular ATP concentration was quantitatively measured. The experimental process is described in detail in Supplemenatry Appendix 6.

### 2.7. Statistics

All data are expressed as the mean ± SD unless otherwise stated. Statistical analyses were performed using SPSS 22.0 (SPSS Inc., Chicago, IL) and GraphPad Prism 6.01 (GraphPad Software, San Diego, CA). Comparisons between two groups were performed using Student's *t*-test, and multiple-group comparisons were performed using one-way ANOVA, followed by Dunnett’s multiple comparisons test where appropriate. Differences were considered significant when *p*≤.05.

## 3. Results

### 3.1. Pi deprivation enhanced DOX intratumoral accumulation and severe necrosis in the VX2 animal model

#### 3.1.1. DOX-TASE in rabbit VX2 liver tumors

**Figure 1.**
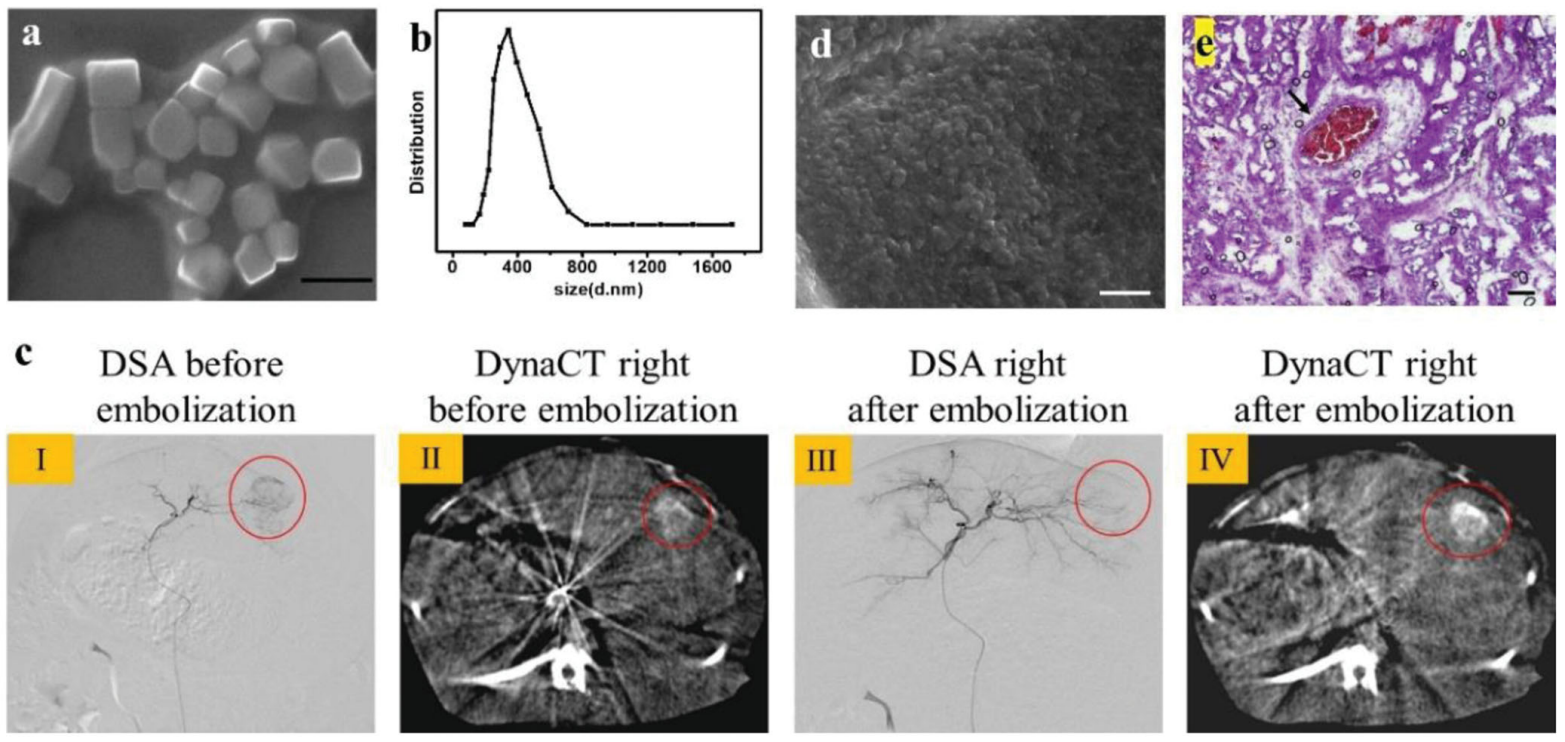
Chemoembolization of VX2 tumors was performed by transcatheter arterial delivery of DOX-sevelamer. (a) Representative SEM image of sevelamer nanoparticles. Scale bar: 1 μm. (b) DLS of sevelamer nanoparticles suspended in Tris–HCl (7.4) buffer solution. (c) Representative DSA and DynaCT images before and after sevelamer embolization (I: DSA before embolization; II: DynaCT image before embolization; III: DSA image right after embolization; IV: DynaCT image right after embolization). (d) Representative SEM image of sevelamer nanoparticles saturated with PBS *in vitro*; scale bar: 1 μm. (e) A typical photomicrograph of H&E staining of slices of VX2 tumors resected immediately after DOX-TASE. The cross-section of the blood vessel in the image center was filled with aggregated sevelamer nanoparticles; scale bar: 1 μm.

After the double milling process, the size of sevelamer particles was reduced from tens of micrometers into polyhedral nanoparticles, and their hydrated radius ranged from 200 to 800 nm with a narrow distribution and positive charge (11.2 ± 0.4 mV), as shown in Figure 1(a,b). DOX mixed with sevelamer nanosuspension was transarterially injected into the tumor-feeding hepatic artery after tumor staining was performed using digital subtraction angiography (DSA) as well as DynaCT (Figure 1(cI,cII)). Under the guidance of DSA, the sevelamer was shown to successfully occlude the tumor blood vessels based on the disappearance of tumor staining (Figure 1(cIII)), and DynaCT immediately performed after embolization showed a high density of iodixanol deposits (Figure 1(b,IV)). These results suggested that sevelamer could effectively embolize tumor blood supply arteries, and the embolization effect predominantly comes from sevelamer aggregation in the presence of endogenous Pi. The anatomical structure of the VX2 liver tumor with a diameter of approximately 15 mm could also be observed after dissection (Figure S1). Vascular embolization by sevelamer nanoparticles might be due to swelling and aggregation induced by endogenous Pi, as shown in Figure 1(d). Sevelamer aggregation within the vessel was observed by H&E staining (Figure 1(e)), indicating successful embolization.

#### 3.1.2. VX2 tumors are characterized by deficient Pi and low levels of ATP and ABC transporters after the DOX-TASE procedure

**Figure 2.**
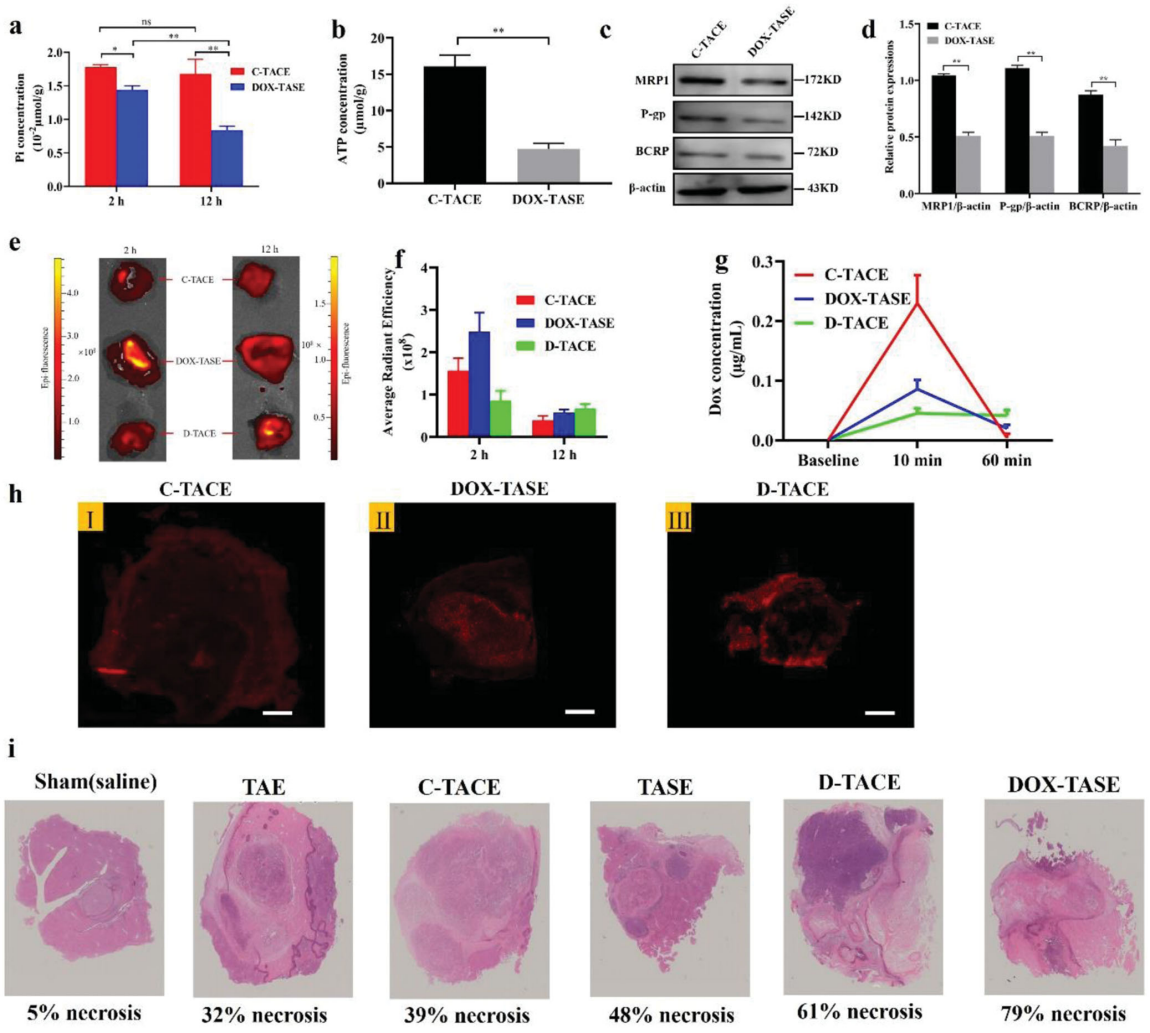
*In vivo* deprivation induced severe necrosis via moderate DOX distribution. (a) VX2 tumors treated with C-TACE or DOX-TASE for 2 h and 12 h were used to measure Pi content, and the same amount of DOX was injected to exclude the effect of DOX on Pi content in tumors. The histogram displays the Pi content after 2 h and 12 h of the two different treatment methods. (b) The histogram presents ATP content after VX2 tumors were treated with C-TACE or DOX-TASE for 2 h. (c) Representative pictures show the WB analysis of BCRP, P-gp, and MRP1 after treatment with C-TACE or DOX-TASE for 2 h. The histogram shows the results of the protein densitometry analysis. One-way analysis of variance (Bonferroni's multiple comparisons test) was used to determine statistical significance. The results are the mean ± SD, *n* = 6, *p<.05, **p<.01. c-TACE vs DOX-TASE. TASE (2 h). (e) Fluorescence imaging of VX2 tumor tissue 2 h and 12 h after treatment with C-TACE, DOX-TASE, and D-TASE. The histogram displays the average fluorescence intensity of each sample. (f) The chart shows the overall fluorescence intensity of the tumor in the three treatment methods. (g) The concentration of DOX in the peripheral blood of VX2 tumor model rabbits treated with C-TACE, DOX-TASE, and D-TACE for 2 min and 60 min was measured by HPLC–MS. The chart presents the changes in drug blood concentration with time in the three treatment methods. (h) Fluorescence scanning image of paraffin sections of tumor tissue from the three groups. (i) Representative histological HE staining of VX2 tumor lesions seven days after embolization. Average necrosis ratio for each group: sham: 8.5 ± 0.7%; TAE: 35.7 ± 2.9%; C-TACE: 40.5 ± 5.3; TASE: 46.9 ± 4.7; D-TACE: 62.3 ± 6.1%; DOX-TASE: 78.9 ± 3.7%.

VX2 tumor rabbits were treated with C-TACE or DOX-TASE, and then the tumor tissues were harvested 2 h and 12 h after the procedure for the determination of intratumoral Pi concentration. No significant change was observed between the two time points after C-TACE treatment, but the intratumoral Pi concentration in the DOX-TASE group significantly dropped to half of the original level 12 h later. Unlike the C-TACE treatment, DOX-TASE treatment deprived the VX2 tumors of Pi (Figure 2(a)).

Two hours after C-TACE or DOX-TASE treatment, the ATP concentration in the VX2 tumor was quantitatively measured. As shown in Figure 2(b), at the same dose of DOX in both groups, the production of ATP in the DOX-TASE group was significantly lower than that in the C-TACE group, suggesting a correlation between Pi starvation and ATP production. The expression of ABC transporters was inhibited in the VX2 tumors treated with DOX-TASE, as evidenced by WB in Figure 2(c–d). Pi starvation led to lower expression of MRP1, P-gp1, and BCRP 2 h after DOX-TASE treatment than that produced with C-TACE.

#### 3.1.3. Pi starvation enhanced the intratumoral retention of DOX and severe necrosis

DOX imaging was undertaken after treatments by using tissue fluorescence analysis to record DOX accumulation dynamics in the VX2 tumor. *Ex vivo* tissue fluorescence imaging results indicated that the fluorescence accumulation of DOX in tumors treated with DOX-TASE was significantly stronger than that in tumors treated with DOX-TACE and D-TACE two hours after the procedure. Twelve hours later, tumors subjected to D-TACE treatment showed the strongest fluorescence intensity, indicative of a slower release of DOX, as expected (Figure 2(e,f)).

HPLC–MS was utilized to measure DOX concentration in the peripheral blood. Whole blood was collected from the femoral artery at 10 min and 60 min after the C-TACE, DOX-TASE, and D-TACE procedures. As shown in Figure 2(g), the plasma DOX level in the C-TACE group was the highest initially (0.217 ± 0.28 µg/mL vs. 0.082 ± 0.012 µg/mL for DOX-TASE, 0.049 ± 0.019 µg/mL for D-TACE). One hour later, the DOX concentrations of tumors treated with D-TACE (0.042 ± 0.007 µg/mL) and DOX-TASE (0.012 ± 0.002 µg/mL) were still measurable; moreover, the DOX concentration of the C-TACE treated tumors had nearly decreased to baseline. The characteristic pharmacokinetics of DOX-TASE seem to be between those of C-TACE and D-TACE.

The fluorescence imaging recorded on the slides for each tumor specimen (at a section through the tumor center) resected 10 min after the procedure showed consistency within the whole tissue, as shown in Figure 2(h). The representative image recorded from C-TACE samples presents the weakest red emission, indicative of low DOX accumulation, but it is technically difficult to tell the fluorescence from interstitial or intracellular DOX due to the limitations of staining the frozen section.

**Figure 3.**
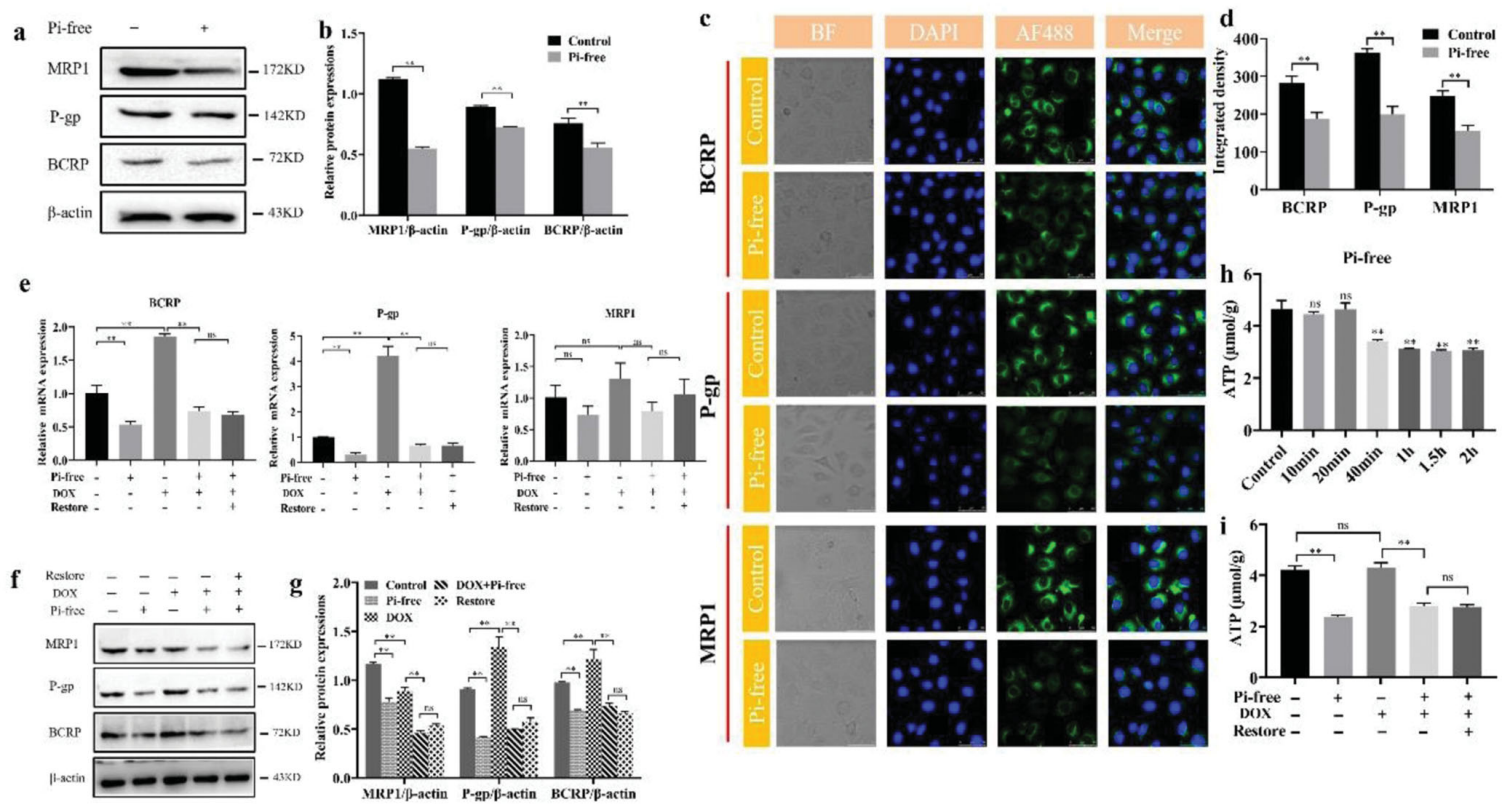
Pi deprivation inhibited the expression of ABC transporter and ATP production *in vitro*. (a, b) Representative WB and statistical analysis of P-gp, MRP1, and BCRP expressed by HepG-2 cells cultured in Pi deprivation medium for 1 h. (c, d) Representative immunofluorescence laser confocal imaging showed the expression of P-gp, MRP1, and BCRP after Pi deprivation for 1 h. (c) mRNA levels of P-gp, MRP1, and BCRP extracted from HepG-2 cells cultured in three conditions were analyzed by Q-PCR, and (f, g) the representative WB plots and the protein grayscale analysis of P-gp, MRP1, and BCRP. All WB results were standardized using β-actin protein. (h) Monitoring of intracellular ATP levels with Pi deprivation against time elapsed. (i) Intracellular ATP level of HepG-2 cells incubated with the different culture conditions. One-way analysis of variance (Bonferroni's multiple comparisons test) was used to determine statistical significance. The values presented are indicative of the mean ± SD (*n* = 6) for each group, **p<.01 vs., ^ns^*p*>.05.

Tumor necrosis outcomes were compared seven days after the procedure and at sacrifice. The necrosis ratio in DOX-TASE-treated samples was significantly higher than that in D-TACE (*p*<.01), C-TACE (*p*<.01), and TASE (*p*<.01) samples, indicating a better therapeutic effect (Figure 3(d)). The typical photomicrographs in the individual groups are listed in Figure 2(i).

### 3.2. Evaluation of Pi starvation-enhanced drug sensitivity in HCC cells

#### 3.2.1. Pi deprivation inhibited the expression of ABC transporter and ATP synthesis

*In vitro* tests were performed on HepG-2 cells, which are human-resourced HCC cells, in Pi-deprived culture medium. As shown in Figure 3(a,b), the protein expression levels of ABC transporters, including P-gp, MRP1, and BCRP, were reduced, as they were in VX2 tumors after the DOX-TASE procedure. Cell immunofluorescence indicated that P-gp, MRP1, and BCRP are expressed on the membrane (Figure 3(c,d)), confirming their role as drug efflux pumps. Their expression quantified by the fluorescent intensity was inhibited under Pi starvation, consistent with the WB results.

The expression of the ABC transporter was regulated not only by Pi stress but also by DOX. As shown in Figure 3(e–g), DOX upregulated the expression of P-gp, MRP1, and BCRP, as confirmed by PCR and WB techniques. The addition of DOX induced HCC cell drug resistance, which was overcome or reversed by Pi starvation. Furthermore, even though the Pi level in the medium was intentionally elevated back to the normal level, the expression levels of P-gp and BCRP were still very low, indicating that the inhibitory effect is irreversible.

Pi deprivation inhibited the production of ATP, as shown in Figure 3(h). Intracellular ATP was quantitatively measured at different timepoints after Pi deprivation, and the production of intracellular ATP significantly decreased 40 min later. DOX had no inhibitory effect on ATP production. This inhibitory effect was also irreversible, as even when Pi in the medium was restored to a normal concentration, no significant elevation in ATP production was observed (Figure 3(i)).

#### 3.2.2. Pi deprivation irreversibly enhances the intracellular accumulation of DOX

**Figure 4.**
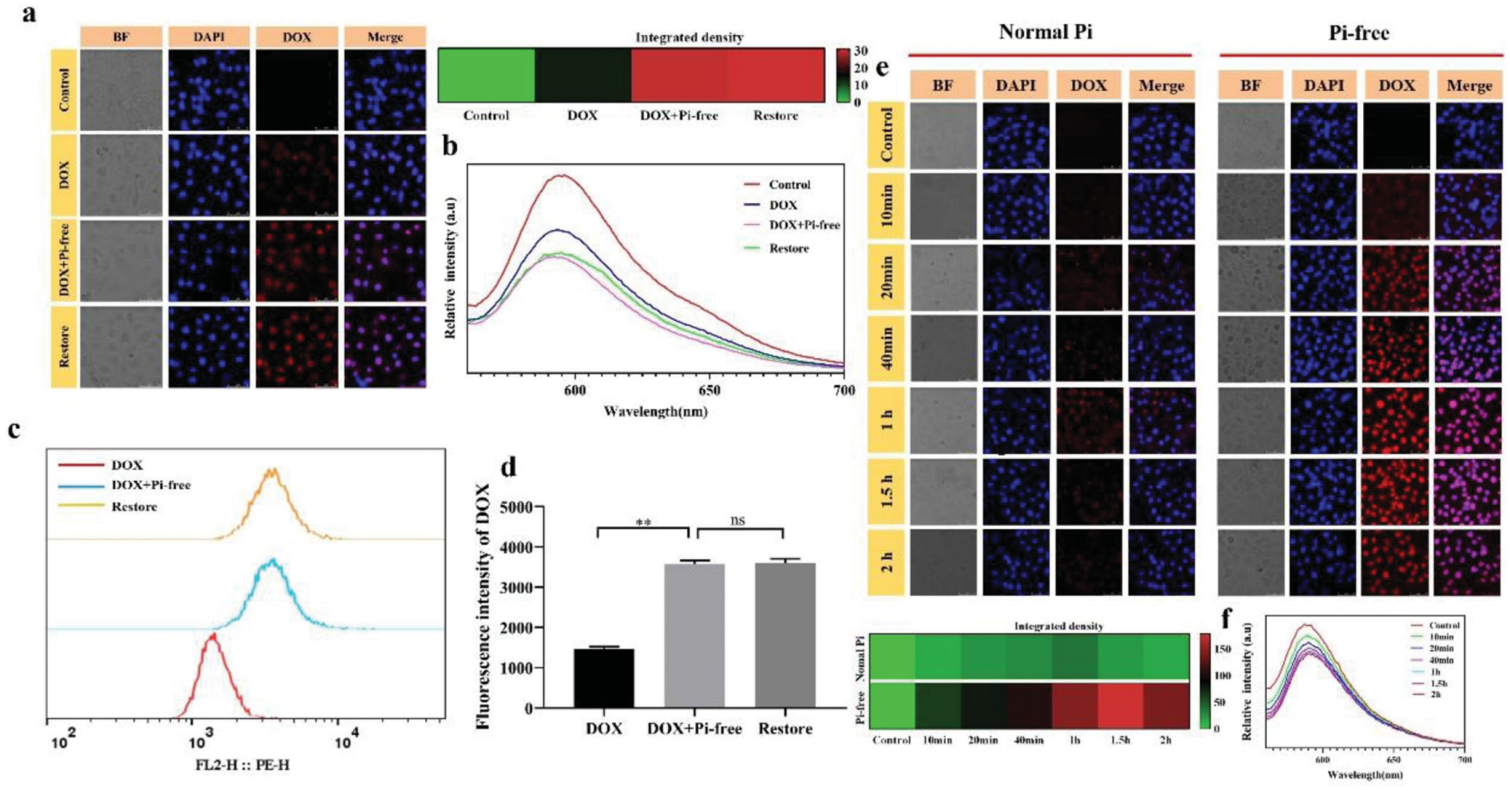
Pi deprivation irreversibly increased the intracellular accumulation of DOX in HepG-2 cells. (a) Confocal microscopic images were recorded 1 h after DOX was co-incubated in Pi-free and normal medium, as well as under sequential Pi starvation and normalization by adding PBS. (b) The fluorescence spectrum recorded the emission from the supernatant of the four samples, indicating that Pi starvation increased DOX intracellular accumulation irreversibly, leaving a lower concentration of DOX in the supernatant. (c) The amount of DOX uptake in HepG-2 cells was determined by FACS. The histogram shows the relative fluorescence intensity of cells treated in each group, and bar graphs represent the relative units ± SEM (standard error of the mean) (*n* = 6). **p<.01, as determined by ANOVA. (e) HepG-2 uptake of DOX was recorded by fluorescence microscopy images at different incubation time points, and the heatmap shows the average fluorescence density. Simultaneously, the fluorescence spectrum of the supernatant was recorded (f).

Pi deprivation inhibited the expression of ABC transporters and the production of ATP; therefore, the influxed DOX has less chance of efflux than that achieved when under normal Pi conditions. As shown in Figure 4, Pi deprivation enhances the intracellular accumulation of DOX. Measurement of the fluorescence intensity of intracellular DOX was used to determine the efficiency of cellular uptake. Under normal incubation conditions, the intracellular DOX concentration was quite low but significantly enhanced upon exposure to Pi-free medium. Pi starvation-dependent DOX accumulation is irreversible since intracellular DOX would not outflow when the Pi concentration in the medium was increased to the normal level. The fluorescence spectra of the supernatants of the four samples also revealed that Pi starvation elevated DOX intratumoral accumulation in an irreversible manner (Figure 4(a,b)). Furthermore, quantitative analysis by flow cytometry (fluorescence-activated cell sorting, FACS) indicated that DOX accumulation within the HepG-2 cells in Pi-free medium occurred irreversibly, as the enhancement did not weaken as Pi in the incubated medium was elevated (Figure 4(c,d)). Time-monitored DOX accumulation under Pi deprivation revealed that the accumulation reached the maximum very quickly by approximately 20 minutes after incubation, and no remarkable influx or efflux was observed after two hours (Figure 4(e,f)). The fluorescence spectra of the supernatants of the four samples showed consistent results.

#### 3.2.3. Pi starvation enhances the drug sensitivity of HepG-2 cells

**Figure 5.**
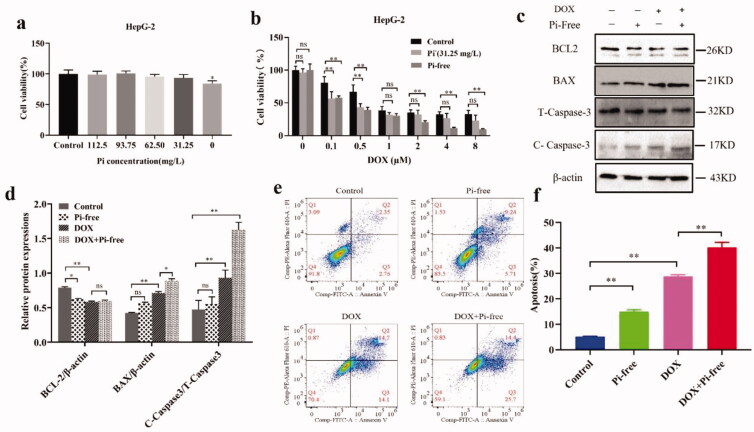
Pi starvation enhanced the apoptotic effect of DOX on HepG-2 cells. (a) The viability of HepG-2 cells was evaluated under various concentrations of Pi (0–125 mg/L) in media for 48 h with MTT assays. (b) HepG2 cells were treated in Pi (–) (31.25 mg/L) and Pi-free (0 mg/L) media supplemented with various concentrations of DOX (0–8 µM) for 48 h, and cell viability was determined with MTT assays. (c, d) The representative chart shows the WB analysis of apoptosis-related proteins (BAX, BCL2, and caspase 3) in HepG-2 cells cultured under different Pi stresses in the presence or absence of DOX for 24 h. The results were standardized using β-actin protein, and the histogram shows the protein gray analysis of different groups. (e, f) Flow cytometry of apoptotic cells and statistical analysis. One-way ANOVA (Bonferroni's multiple comparison test) was used for analysis to determine statistical significance. The data are expressed as the mean ± SD, *n* = 3, *p<.05, **p<.01 vs. control. FITC: fluorescein isothiocyanate; PI: propidium iodide.

**Figure 6.**
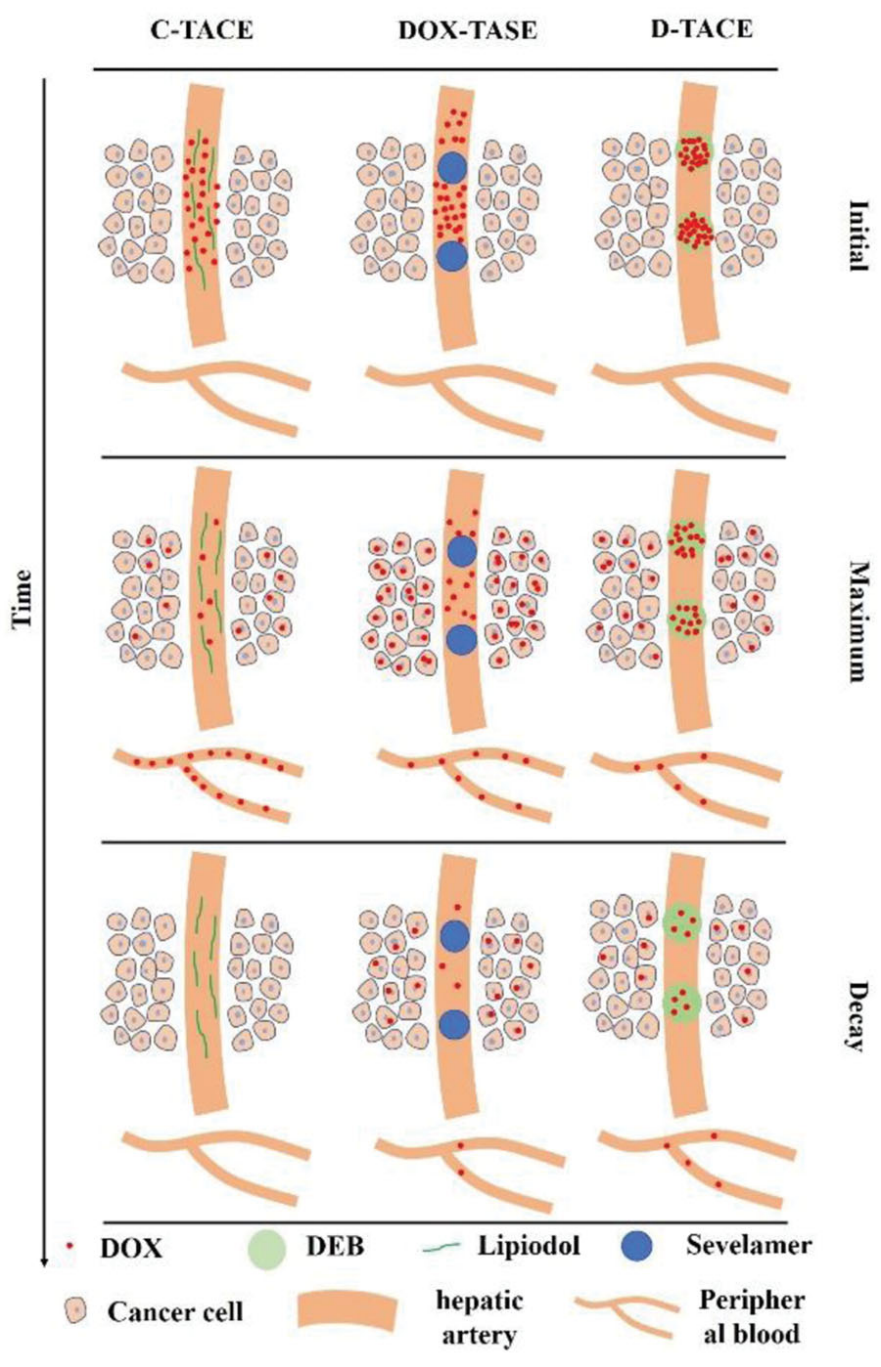
Schematic of the dynamic distribution of DOX in the artery, intracellularly, and in peripheral blood.

HepG-2 cells were cultured in different Pi concentrations (0–125 mg/L) for 48 h. The MTT assay showed that only Pi was completely exhausted, and proliferation was remarkably inhibited, as shown in Figure 5(a). The Pi concentration even dropped to 31.25 mg/L, one-quarter of the normal Pi concentration value (125 mg/L), indicating that Pi starvation itself did not affect the proliferation of HepG-2 cells over a wide range. HepG-2 cells proliferated under different DOX concentrations (0–8 μM) for 48 h under three types of Pi concentrations, including normal (125 mg/L), threshold (31.25 mg/L), and Pi-free (0 mg/L) states, and the changes in cell activity induced by DOX were measured under the different Pi concentrations. As shown in Figure 6(b), under normal Pi concentrations, the cell activity was significantly inhibited with increasing DOX concentrations (IC_50_=0.972 μM). In contrast, when Pi was reduced to a lower level, HepG-2 cells became more sensitive to drugs (IC_50_=0.210 μM for 31.25 mg/L; IC_50_=0.196 μM for 0 mg/L) (Figure 5(b)). The IC_50_ values of DOX treatment under the latter Pi conditions (0.210 μM vs. 0.196 μM) were very close, indicating that the cytotoxicity predominantly comes from anticancer drugs and is not directly related to the Pi environment.

Cancer death induced by DOX is closely associated with apoptosis, as shown in Figure 5(c,d). Apoptosis-relevant proteins (BAX, BCL2, and caspase 3) were expressed in a Pi-dependent manner. There was a significant increase in levels of BAX and cleaved caspase-3, but the expression of BCL2 was significantly downregulated, indicating that Pi starvation has a synergistic effect on cell apoptosis. Flow cytometry supplied the same results (Figure 5(e,f)). Both results suggested that Pi deprivation could promote the apoptosis of HepG-2 cells triggered by DOX.

## 4. Discussion

Based on the concept of Pi deprivation as a new anticancer strategy, we have proposed TASE as an alternative embolization technique to treat HCC in the rabbit VX2 model (Bi et al., 2022). Herein, we utilize this strategy to overcome and reverse drug resistance to enhance TACE therapy via promoting the increased accumulation of intracellular DOX. Considering the results of *in vivo* and *in vitro* tests, we can deduce the drug distribution process, as shown in Figure 6. Initially, the same dose of DOX was transarterially delivered with different media or carriers: lipiodol for C-TASE, sevelamer for DOX-TASE, and microbeads for D-TACE, where DOX was incorporated into the framework with electrostatic interactions or other noncovalent bond forces. DOX exhibits different fates across the various groups. Due to the high mobility of lipiodol, DOX quickly washes out into the peripheral vessel, and it has less chance to diffuse into the interstitial space, let alone the intracellular space. D-TACE delays the release of DOX; therefore, even months later, DOX remained in the patients’ plasma, and VX2 rabbits showed consistent results. D-TACE resolves the problem of wash out exhibited in C-TACE (Varela et al., 2007); however, the issue of the efficiency of DOX uptake by HCC cells is ignored but is the most critical, although incremental increases in DOX were found to correlate with greater necrosis (Gaba et al., 2016), as well as better concurrent chemoradiotherapy (Wang et al., 2021). As evidenced in the *in vitro* tests, Pi starvation causes HCC cells to uptake DOX with higher accumulation and longer retention time. Unfortunately, it is difficult to distinguish intracellular from interstitial DOX due to the complexity of slice processing.

*In vitro* tests indicated that Pi starvation enhanced the intracellular accumulation of DOX via inhibition of the expression of ABC transporters and the production of intracellular ATP. Furthermore, *in vivo* tests confirmed that upon intratumoral Pi deprivation, DOX-TACE showed a better therapeutic effect than that of C-TACE and D-TACE. Thus, Pi starvation reverses DOX resistance in HCC, both *in vitro* and *in vivo*, as we demonstrated. DOX-based chemoembolization is a key therapy for intermediate HCC, but its utility is limited by preexisting and acquired tumor resistance, as we proved that DOX itself also enhanced drug resistance by upregulating the expression of ABC transporters. The mechanisms of DOX resistance include increased expression of multidrug resistance efflux pumps, alterations of the drug target, topoisomerase activity, and modulation of programmed cell death pathways. Clinical resistance of HCC to DOX involves multiple mechanisms largely related to changes in drug accumulation, apoptotic signaling, or topoisomerase activity (Cox & Weinman, 2016). It is also the main reason responsible for equivalent efficiency between TAE and TACE, and even D-TACE has no advantage over C-TACE, although DOX was released from DEB for months. Most anti-HCC drugs require certain intracellular levels to be reached and maintained for enough time, so any change resulting in a reduction in intracellular drug accumulation may compromise treatment success (Marin et al., 2020).

The conventional method used to downregulate the expression of ABC transporters and reverse drug resistance in HCC is to introduce novel drugs, including cantharidin, glycyrrhizin and lamivudine, resveratrol and levistolide (Wakamatsu et al., 2007; Zheng et al., 2008; Kim et al., 2014; Ding et al., 2019). Our investigation was undertaken to resolve this unsettled issue by utilizing the local-regional technique to change the tumor chemo-environment. We try to address drug resistance by a subtraction strategy, instead of through addition as is normally done. Since chemoembolization therapy temporarily cuts off the blood supply from the artery, the tumor is isolated for a longer or shorter time, depending on the different embolic agents. This isolation starves cancer cells from nutrients and oxygen, thereby changing the tumor chemical microenvironment for some time and inducing features such as severe hypoxia, which is considered a critical promoter of tumor recurrence and drug resistance (Petrillo et al., 2018; Bao & Wong, 2021). The advantage is that the embolotherapy technique can also be utilized to create a new chemical environment, such as through inducing Pi starvation. In this particular chemo-microenvironment of low Pi stress, the relationship between Pi starvation, the amount of DOX retention, and the tumor necrosis ratio was evidenced.

All the *in vitro* tests identified that Pi starvation irreversibly increased the cellular influx of DOX as the expression of three common MDR plasma membrane proteins driving the efflux of drugs (P-gp, BCRP, and MRP1) was effectively impaired as Pi stress led to downregulated expression levels of ABC transporter genes, which has been reported to influence the efficacy of chemotherapy in patients with HCC (Korita et al., 2010). As we proved, those MDR proteins are less expressed in the Pi-deprived environment, and ATP is also less produced, which is consistent with our recent findings evidenced by metabolomics (Bi et al., 2022). In other words, there is a lack of both ‘vehicles’ and ‘gas’, and intracellular drugs cannot be passively transported out and must remain inside the cell. Enhanced intracellular uptake of DOX *in vitro* results in improved antitumor activity *in vivo* (Xiong et al., 2005; Marin et al., 2018), as we proved that necrosis in the DOX-TASE group is much higher than that in the DOX-TACE or pristine TASE group. Although the mechanism of drug resistance might come from eight possible pathways, DOX resistance in HCC is also being considered with multiple pathways (Cox & Weinman, 2016; Bar-Zeev et al., 2017). Pi starvation at least opens two strategies for resolving the resistance.

The irreversibility of Pi starvation-dependent DOX reflux is also very important since TASE only guarantees a short period of intratumoral Pi starvation, which will be compensated for by the blood fluid. However, Pi compensation does not change the anti-resistance property of Pi starvation. This irreversibility is an important MDR phenotype caused by Pi starvation since after sevelamer-mediated chemoembolization, Pi starvation within the tumor might not persist for a long time, and Pi outside the tumor will diffuse gradually; however, fortunately, efflux of DOX will not occur in response to the elevation of Pi concentration, thereby guaranteeing the drug’s long intracellular retention time.

High levels of inorganic phosphate have been reported to promote tumor progression via multiple pathways which do not include promotion of drug resistance (Brown & Razzaque, 2016, 2018). Although we could not identify the relationship between high Pi levels and drug resistance, we found that low Pi levels helped to overcome drug resistance in HCC cells. Drug resistance is very complex and modulated by genetic and epigenetic alterations that affect drug uptake, metabolism, and export of drugs at the cellular level. As chemical characteristics of the tumor microenvironment, hypoxia and acidity influence the sensitivity of the tumor cells to drug treatment (Tredan et al., 2007), and we have found that low Pi stress induces some anticancer effects (Bi et al., 2022). Therefore, intratumoral Pi starvation might inhibit or retard those pathways beneficial for drug resistance. TASE would be a helpful strategy for increasing the efficacy of DOX by promoting intracellular accumulation of DOX and decreasing multidrug resistance in HCC cells.

There are some shortcomings to this study. Clinical resistance of HCC to DOX involves multiple mechanisms largely related to changes in drug accumulation, topoisomerase activity, or apoptotic signaling (Cox & Weinman, 2016). Pi starvation might not only resolve the issue of drug accumulation, but more studies are ongoing. Second, systemic toxicity of this approach has not been evaluated, which might impede its clinical application (Zheng et al., 2021). Finally, this strategy is limited to treating solid tumors adaptable for chemoembolization therapy, such as HCC.

## 5. Conclusions

DOX-based chemoembolization is a key therapy for HCC treatment, but its utility is limited by preexisting and acquired tumor resistance. Therapeutic approaches to overcome resistance are an important future goal but have not yet reached clinical practice. Herein, we present a Pi starvation strategy to reverse drug resistance in HCC, as evidenced by a series of *in vitro* and *in vivo* tests, thereby benefiting chemoembolization therapy. As DOX-mediated TACE is easy to perform for interventional clinicians, this strategy is highly translational to hepatic interventional oncology in the future.

## Supplementary Material

Supplemental Material
