# Supplementary material for: Intratumoral Pi deprivation benefits chemoembolization therapy via increased accumulation of intracellular doxorubicin
Source: Drug Deliv. 2022 May 30;29(1):1743–53. doi: 10.1080/10717544.2022.2081384 (PMC9176673; doi:10.1080/10717544.2022.2081384)
Supplement: Supplemental Material [file IDRD_A_2081384_SM2060.docx]

**Appendix 1**

The methods for VX2 tumor development and liver tumor generation have been described previously [1]. Briefly, frozen rabbit VX2 tumor samples were defrosted and injected into the hind limb muscle of donor New Zealand white rabbits for incubation. Approximately 2–3 weeks after implantation, the donor rabbits were sacrificed, and the hind-limb tumors were excised and transected, after which several pieces of 1mm3 tumor were selected for liver implantation. For these surgeries, recipient rabbits were medicated for anesthetic induction with ketamine and dexmeditomidine, followed by intubation and maintenance with 1%–3% isoflurane. One preprocedural dose of enrofloxacin (5 mg per kilogram of body weight, administered subcutaneously) antibiotic prophylaxis was provided. Under aseptic conditions, a mini-laparotomy was performed in the subxiphoid area, exposing the liver. Tumor fragments of 1 mm3 were freshly harvested from donor rabbits and implanted in the left hepatic lobe of recipient rabbits, and then inoculated with puncture needle in the liver parenchyma. A cotton swab was utilized to stop bleeding by pressing the puncture point. The abdomen was then safely closed. After the procedure, the animals were aroused and recovered, returned to their cages, and monitored daily for wound healing and appetite until TACE procedure. Liver tumors were incubated for 2 weeks prior to TACE based on the experience of suitable 1-2 cm diameter tumor growth within 14 days.

**Appendix 2**

Two weeks after implantation, general anesthesia was induced and a surgical cutdown was performed to gain access into the right common femoral artery for vascular access, after which a 4-F vascular sheath (Cook, Bloomington, IN) was placed, and a 4-F catheter (Cordis, Miami Lakes, FL) was inserted into the aorta. Then another 1.98-F catheter (ASAHI INTECC, ASAHI INTECC CO., LTD) was manipulated into the celiac axis and the hepatic artery or superior mesenteric artery feeding the tumor through a 0.018-inch diameter guide wire (Asahi Intec, Hanoi, Vietnam). digital subtraction angiography (DSA) procedures equipped with DynaCT (Artis Zee Ceiling, SIEMMENS) were performed by an attending interventional radiologist (R.-G. Luo, with more than 10 years of VX2 TACE experience). Different agents in various cohort were injected carefully. After that, the catheter was removed, and the common femoral artery was ligated using resorbable suture material to obtain hemostasis. The animals were returned to cages and followed-up daily until their respective times of sacrifice, or regular blood sampling.

**Appendix 3**

Sevelamer hydrochloride (Renagel; hereafter referred to sevelamer) was purchased from Wuhan Hezhong Pharmaceutical Co., Ltd, and sevelamer nanoparticles were prepared via a two-step gridding technique. As a microcrystalline powder, its size is around several hundreds of micrometers with an irregular shape. Sevelamer (2 g in 30 mL DI water) was mixed with inert balls (Φ15mm: Φ5mm: Φ2mm: Φ0. 5mm = 50g: 50g: 50g: 50g) in the autoclave, ground at the speed of 300 RPM for 20 h in 30 ml water in QM-3SP4 planetary ball mill (Nanjing Nanda Instrument Co., Ltd.). The products were separated from the inert balls and re-milled (Φ0.05mm=200g) in 30 ml water for 6 h. The gridded sevelamer was lyophilized and stored at room temperature. The hydrated diameter and the surface charge of sevelamer were measured by dynamic light scattering technique (DLS) (Malvern R-Zetasizer Nano ZS90) after dispersion in Tris–HCl buffer (pH 7.4). Note that the particle size and charge of sevelamer nanoparticles were calculated as an average of triplicate measurements. All the statistical data are expressed as mean ± SD.

A scanning electron microscope (FEI Quanta200F) was applied to record in vitro Pi-activated swell and aggregation of sevelamer nanoparticles. In detail, phosphate-buffered solution (PBS) containing phosphate mass equivalent to the full phosphate binding capacity (5.5 mmol/g) was mixed with sevelamer for 30 minutes at 37^o^C. The unreacted, and saturated sevelamer were imaged after lyophilization and Au evaporation.

**Appendix 4**

Ultra-high performance liquid chromatography (UHPLC) separation was performed on LC30 system UPLC system (Shimadzu, Japan), and the analytical column used was SHIM-PACL GIST C18 (2.1 × 75 mm，1.8 μm). Separation was performed using 0.1% formic acid water (Solvent A) and 0.1% formic acid acetonitrile (Solvent B). The mobile phase flow rate was set to 0.3 ml / min, the column temperature was 35 ^o^C, and the injection volume was 5 µL.

UHPLC system is coupled with hybrid quadrupole TOF mass spectrometer system (AB SCIEX) in positive ion mode. Data was acquired by single reaction monitoring (SRM) mode. The capillary voltage is 3.8 kV. The source block and desolvation temperatures are set to 150 ^o^C and 500 ^o^C, respectively. The conical gas flow is 50 L / h and the desolvation gas flow is 650 L / h. Argon is used as a collision gas. The ion pairs used for the quantitative SRM method are 544.0 to 397.0 (DOX).

100 µ l of serum was added to 1 ml of cold organic phase (chloroform: methanol = 4:1) and vortex mixed for 4 minutes. The mixture was centrifuged at 12000 rpm at 4 ^o^C for 5 minutes. Transfer the supernatant to a clean micro centrifuge tube, repeat this operation twice, mix, collect and dry the supernatant. The samples were recombined in 100 µ L. Then inject an aliquot of 5 µ l into the MS device for analysis.

**Appendix 5**

The tumor tissue with the same weight (50mg) was placed in saline (1mL) and homogenized with a tissue homogenizer. Collected the supernatant after centrifugation (12000rpm, 10min), and the mass concentration of inorganic phosphate (Pi) in the tumor at different time points was detected by phosphomolybdic acid colorimetry.

**Appendix 6**

The detection of ATP is based on the need for ATP to provide energy when firefly luciferase catalyzes luciferin to produce fluorescence. When firefly luciferase and luciferase are in excess, the production of fluorescence is directly proportional to the concentration of ATP in a certain concentration range, so that we can detect the concentration of ATP in cells with high sensitivity. The ATP content was measured using an ATP assay kit (Beyotime, China).

For tumor tissue samples, added about 500 µl lysis buffer to per 50 mg tissue, and then used a glass homogenizer for homogenization. Sufficient homogenization could ensure that the tissue was completely lysed. After lysis, centrifuge at 12,000g for 5 minutes at 4°C, and take the supernatant for subsequent determination.

For cells, according to the instruction, cells were lysed and centrifuged at 12,000×g for 5 min. The supernatant was collected and mixed with the ATP detection working dilution in a ratio of 1:9. The luminescence intensity was detected by a microplate reader (Bio-Rad, Microplate Reader 550). The results were standardized by the molar mass of ATP per milligram of protein.

**Appendix 7**

Cells or tissues were lysed in lysis buffer containing 10% PMSF and cultured on a shaking table at 4 ^o^C for 30 minutes. The supernatant was collected after centrifugation at 12000rpm for 10 minutes. Subsequently, the protein concentration was measured by the double golden Cinna acid (BCA) protein analysis kit (Beyotime, China). About 20 g protein was separated by electrophoresis on twelve alkyl sulfate polyacrylamide gel, and then transferred to Immobilon FL polyvinylidene fluoride (PVDF) film (Merck, Germany). After 5% skimmed milk was sealed on the shaking table at room temperature for 1h, the membrane was incubated with primary antibody diluted at 1:1000 at 4 ℃ overnight, and then incubated with secondary antibody at room temperature for 1H. Washed the membrane three times with Tris-buffer saline contained 0.1% Tween 20 (TBST). Finally, the signal of the film was detected by enhanced chemiluminescence (ECL). The main antibodies used in this study include Anti-MRP1, Anti-MRCP, Anti-P-gp, Anti-β-Actin, Anti-BCL2, Anti-BAX, and Anti-Casepase3. For quantitative analysis, the protein expression level was standardized to the corresponding level β-Actin levels.

**Appendix 8**

Doxorubicin hydrochloride was purchased from Macklin (Shanghai, China). Sevelamer hydrochloride was purchased from Wuhan Hezhong Pharmaceutical Co., Ltd (Wuhan, China). Microculture tetrazolium (MTT) was purchased from Beyotime (Shanghai, China). Cell culture medium and supplements, including antibiotics and fetal bovine serum (FBS), were obtained from GIBCO (NY, USA). Primers for reverse transcriptase-polymerase chain reaction (RT-PCR) were synthesized by Takara PCR Thermal Cycler Dice (Shiga, Japan). Antibodies against MRP1, P-gp, BCRP, BCL2, BAX, Casepase3, and β-actin were purchased from Abcam (Cambridge, UK). All other chemical reagents were purchased from Sigma-Aldrich (St. Louis, MO, USA).

For all experiments, the Doxorubicin hydrochloride was dissolved in DMSO (mg/mL) to prepare a stock solution, and the final concentration of the Doxorubicin hydrochloride was obtained by diluting the stock solution in Dulbecco's Modified Eagle Medium (DMEM) medium with different Pi concentration, including normal Pi concentration (125mg/L), low Pi or Pi-free medium. Among them, the low Pi medium or Pi-free medium was obtained by adding different mass Sevelamer Hydrochloride (phosphate-binding rate was 5.5mmol/g) to the normal Pi concentration medium and then filtered through a 0.22μm filter. The final concentration of DMSO was less than 0.1% to avoid cytotoxicity.

Human-resourced liver cancer cells (HEPG-2) and VX2 cells were purchased from the Cell Bank of the Chinese Academy of Sciences (Shanghai, China). Cells were cultured and maintained in DMEM medium (BI, Israel) enriched with 10% FBS (BI, Israel) and 100 units/mL of penicillin-streptomycin (Solarbio®, China) in a 5% CO2 humidified incubator at 37 °C.

**Appendix 9**

The Q-PCR primers used in this study are listed in the table.

| Primer name | Primer sequence (5’-3’) |
| --- | --- |
| RT-P-gp-F  RT-P-gp-R  RT-MRP1-F  RT-MRP1-R  RT-BCRP-F  RT-BCRP-R  RT-β-Actin-F  RT-β-Actin-R | CCCATCATTGCAATAGCAGG  GTTCAAACTTCT GCTCCTGA  ATCAAGACCGCTGTCATTGG  TCTCGTTCCTACTGAACGTC  TGCCCAGGACTCAATGCAAC  ACAATTTCAGGTAGGCAATT  AGGCACCAGGGCGTGAT  GCCCACATAGGAATCCTTCTGAC |


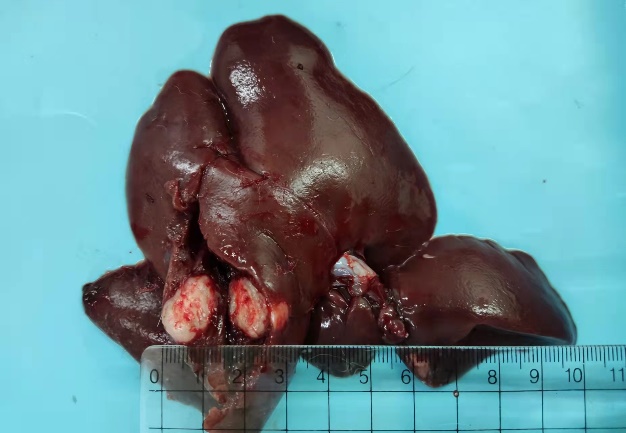


Figure S1. A typical VX2 tumor implanted in the left lobe of the rabbit liver.

[1] Parvinian A, Casadaban LC, Gaba RC. Development, growth, propagation, and angiographic utilization of the rabbit VX2 model of liver cancer: a pictorial primer and “how to” guide. Diagn Interv Radiol 2014;20(4): 335–340.
